# Forward Light Scattering of the Vitreous Gel After Enzymatic Aging: An In Vitro Model to Study Vitreous Opacification

**DOI:** 10.1167/iovs.65.3.36

**Published:** 2024-03-29

**Authors:** Maximilian Hammer, Marcel Muuss, Sonja Schickhardt, Alexander Scheuerle, Ramin Khoramnia, Grzegorz Łabuz, Philipp Uhl, Gerd Uwe Auffarth

**Affiliations:** 1 University Eye Clinic Heidelberg, Heidelberg, Germany; 2 The David J Apple Laboratory for Vision Research, Heidelberg, Germany; 3 Institute for Pharmacy and Molecular Biotechnology, Heidelberg, Germany

**Keywords:** vitreous body, straylight, forward light scattering, floaters, opacification

## Abstract

**Purpose:**

Symptomatic vitreous opacifications, so-called floaters, are difficult to objectively assess majorly limiting the possibility of in vitro studies. Forward light scattering was found previously to be increased in eyes with symptomatic floaters. Using an objective setup to measure forward light scattering, we studied the effects of enzymatically digesting the components of the vitreous body on straylight to develop an in vitro model of vitreous opacifications.

**Methods:**

Fifty-seven porcine vitreous bodies were digested using hyaluronidase, collagenase, trypsin, and bromelain, as well as using a combination of hyaluronidase + collagenase and hyaluronidase + bromelain. A modified C-Quant setup was used to objectively assess forward light scattering.

**Results:**

Depletion of hyaluronic acid majorly increased vitreous straylight (mean increase 34.4 deg^2^/sr; *P* = 0.01), whereas primarily digesting the vitreous gel with collagenase or trypsin did not significantly affect straylight. When collagenase or bromelain is applied in hyaluronic acid depleted vitreous gels, the increase in forward light scattering is reversed partially.

**Conclusions:**

The age-related loss of hyaluronic acid primarily drives the increase in vitreous gel straylight induced by conglomerates of collagen. This process can be reversed partially by digesting collagen. This in vitro model allows the objective quantification and statistical comparison of straylight burden caused by vitreous opacities and, thus, can serve as a first testing ground for pharmacological therapies, as demonstrated with bromelain.

With advancing age, age-related changes in the vitreous body occur, such as liquefaction (synchysis) and fiber aggregation (syneresis). Owing to conformational changes in the interaction between hyaluronic acid and collagen, the main components of the vitreous body (Fig. 1) become increasingly dysfunctional. This process results in areas with a higher concentration of aggregated collagen fibers and regions without fibers containing liquefied gel, known as lacunae.^1^^–^^3^

**Figure 1.**
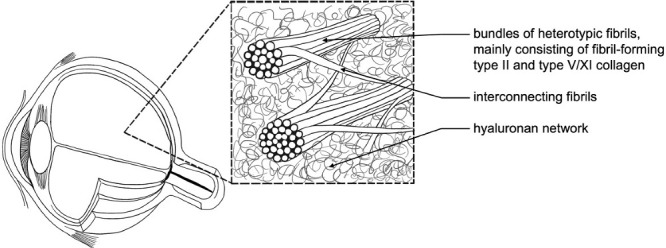
Schematic illustration of the three-dimensional structure of the vitreous. Heterotypic collagen fibrils, including collagen types II, V/XI, and IX, are organized into small bundles that are interconnected by fibrils reaching from one bundle to another. This network forms the basis for a highly hydrated (>98% water) gel structure. The spaces between the collagen fibrils are filled with glycosaminoglycans, which, except for the predominant glycosaminoglycan hyaluronic acid, are bound to a protein core, such as type IX collagen, forming proteoglycans.^3^^,^^10^

The aging of the vitreous body is a leading cause of the emergence of eye floaters, also known as *mouches volantes*.^4^ These floaters arise because of deposits consisting of protein or cell debris that are located in pockets of liquid within the vitreous body or between the vitreous and the retina.^5^ In some cases, they become visible because of the shadow cast on the retina or the refraction of light passing through them, even if the opacity is transparent.^6^ They may look like blobs, little worms, or cobwebs and float slowly through the observer's visual field moving in the direction of the eye movement.^7^ Although floaters are a common entoptic phenomenon, also in young eyes, and typically harmless and unnoticed, they can, in some severe cases, be highly bothersome and have a significant impact on a person's quality of life.^8^^,^^9^

Floaters acquired later in life may diminish or disappear within weeks or months. In these cases, psychological as well as physical parameters are suspected to play a role.^11^ However, in severe cases of floaters, radical therapy may become necessary. Currently, there are primarily two therapeutic options, namely surgery^12^^,^^13^ and laser treatment.^14^^,^^15^ Vitrectomy, the surgical removal of the vitreous body, has a high success rate in relieving the burden of floaters.

Nevertheless, this serious intervention may lead to accelerated cataract development and is associated with potentially sight-threatening complications such as retinal detachment.^16^ Another option is the thermal fragmentation of vitreous opacities using a YAG laser.^14^ The targets need to be identifiable by the treating physician, which may be challenging owing to the little amount of backscattered light.^17^ Compared with surgical options, this method has multiple downsides, with a lower success rate and the development of new floaters following the fragmentation. Additionally, it carries a high risk of damaging the surrounding ocular tissue.^18^ However, a promising improvement of this therapy using nanobubble ablation was recently introduced by Sauvage et al.^19^

Compared with these approaches, a noninvasive pharmacological treatment for floaters could be a breakthrough. Fruit enzymes such as bromelain, papain, and ficin^20^ are discussed to have a positive impact in reducing floaters.^21^ In 2022, a controversial clinical study from Taiwan^22^ claimed that a daily oral application of these enzymes in a high dose could cause a disappearance rate of spontaneous symptomatic vitreous opacities of up to 70% within 3 months.

The goal of this study was to assess if the enzymatic degradation of the structure of the vitreous body via intravitreal injections could serve as a model to evaluate floater therapies, such as bromelain, in the future. Thus, we injected previously established concentrations of hyaluronidase, collagenase, trypsin, bromelain, and combinations of these enzymes to establish their impact on vitreous straylight. Although viscoelastic changes in the vitreous body with age, like the decrease in viscoelasticity,^23^ are well-analyzed, there is little to no literature on the optical parameters of the vitreous body yet. One previous study^17^ showed that forward light scattering is increased in eyes with symptomatic floaters. Thus, straylight might be a reliable objective parameter for the burden of vitreous opacifications. Eyes with enzymatically treated vitreous gels to increase straylight could then serve as a model for eyes with floaters. Therapeutic interventions could therefore be evaluated and quantified based on the effect on forward light scattering.

## Methods

### Vitreous Body Preparation

Fresh porcine eyes were obtained from Schradi Frischfleisch (Mannheim, Germany) and transported to Heidelberg University (<1 hour). After enzymatic degradation, a small incision was made approximately 5 mm posterior the corneal limbus, and scissors were used to enlarge the incision. The vitreous body together with the crystalline lens, pigmented tissue of the ciliary body, and the iris could then be moved gently to a sterile Petri dish. Using a forceps, the vitreous gel could now be grasped in its anterior region and lifted up. Subsequently, the lens and pigment were removed carefully. The vitreous gel was then gently rinsed with balanced salt solution to remove any pigment or retinal debris and then placed directly into the optical measurement cuvette. This relocation of the vitreous gel is needed to allow consistent and objective measurements of forward light scattering. Currently, no methodology is available that allows in vivo measurements of the isolated contribution of the vitreous to ocular straylight. The relocation causes the partial loss of the liquid part of the vitreous body. A total of 57 vitreous gels were measured in this study. Owing to the large number of measurements to be conducted, measurements were split into a total of five sessions. Individual eyes could not be paired and were assigned randomly and, because every measurement session had to be performed with fresh porcine eyes, measures to compare the baseline straylight of different porcine eye shipments had to be in place. For all sessions, multiple eyes were sham injected with balanced salt solution and their straylight was evaluated at the same time as the enzymatically digested vitreous gel. These baseline straylight values were subtracted from the straylight measurements of the enzymatically digested vitreous gels for this specific measurement session. To minimize baseline differences, all eyes were received from the same slaughterhouse which only handles pigs aged 8 to 9 months from the same breeders in the region of Baden-Württemberg, Germany.

### Forward Light Scattering Measurements

**Figure 2.**
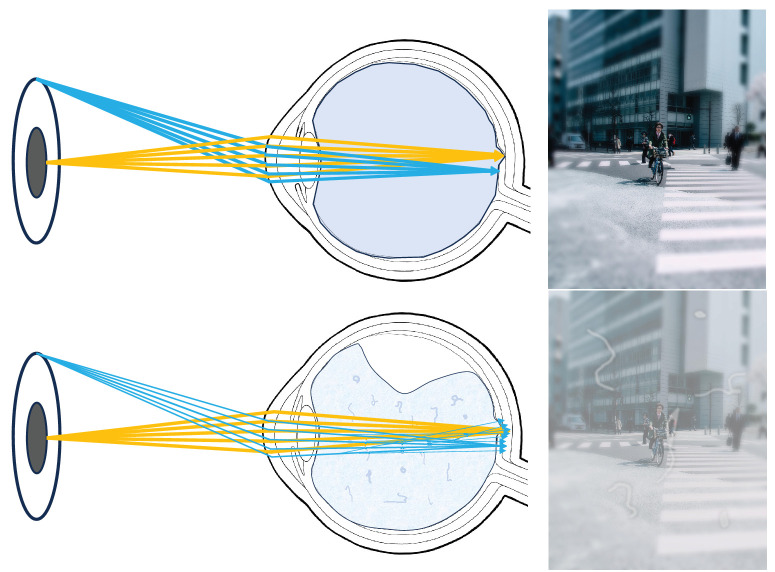
Potential impact of vitreous aging on forward light scattering. With advancing age, the vitreous body becomes dysfunctional (*bottom*). The interfibrillar distance decreases; thus, forward light scattering and the phenomenon of floaters may increase. Patients may notice changes in vision such as decreased contrast sensitivity, glare, and straylight.

**Figure 3.**
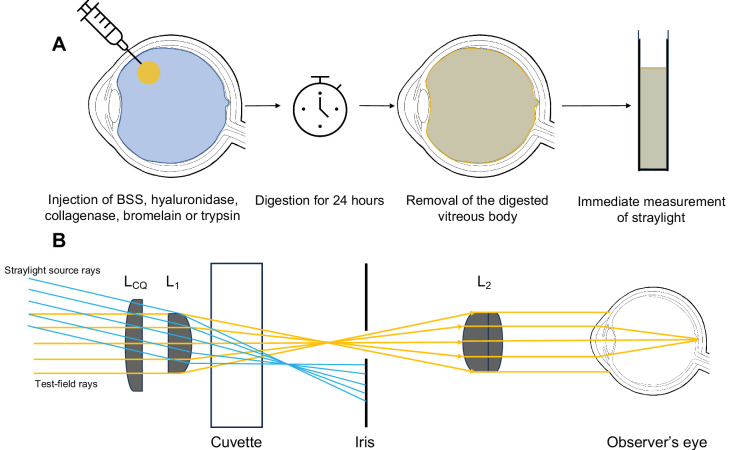
Schematic illustration of the study protocol and in vitro forward light scattering measurements. (**A**) First, nondamaged porcine eyes were injected with either saline or an enzymatically active component. After a digestion period of 24 hours, the vitreous gel was carefully removed from the vitreous cavity, washed with balanced salt solution and then placed in a cuvette to undergo forward light scattering measurements. (**B**) Figure modified from Labuz et al.^25^ Schematic illustration of the C-Quant adaptation used to objectively measure forward light scattering in vitro. The setup consists of two lenses (L_1_ and L_2_) in addition to the C-Quant lens (L_CQ_). The iris acts as a field-stop intercepting the rays that come from the straylight source while the test field can still be seen. L_2_ assists by magnifying the test field. Thus, only the straylight induced by the substance in the cuvette is objectively quantified. This approach was favored over a direct measurement owing to a more confined passage of rays through a test sample, and thus, having a lower potential for creating edge effects.

For the measurement of the straylight a methodology, which was first introduced by van den Berg et al.^24^ to measure ocular straylight clinically, was modified by our group for light scattering measurements.^25^^–^^27^ In essence, a repeatable and objective evaluation of straylight becomes possible with the help of an additional custom-made optical mount for the C-Quant (Oculus, Wetzlar, Germany), a straylight meter for diagnostical purposes. Figure 2 illustrates the concept of in vivo straylight measurement. The C-Quant works on the basis of a psychophysical compensation comparison method and the added mount enables the measurement of the vitreous gel's straylight only. The mount consists of lenses and a field-stop setup that prevents the straylight source from being visible to the observer. This process enables the observer only to judge the light scattered by the measured substance in the measurement cuvette without any contribution to the straylight of the observer's eye. The straylight induced by the whole setup (e.g., the cuvette and the correcting lenses) must be subtracted from the measured straylight. This baseline straylight was measured using the same cuvette setup filled with a balanced salt solution instead of components of the vitreous gel. Each vitreous gel sample was measured three times in a row by the authors, confirming the consistency of our results (M.H., M.M.). The in vitro measurement setup is depicted in Figure 3B.

### Enzymatic Degradation

Based on the work of Filas et al.,^28^ structural macromolecules were digested enzymatically in a subset of eyes before dissection. The following enzymes were chosen because they specifically target the most important structures of the vitreous body: trypsin (1 mg/mL in injection), hyaluronidase (0.625 mg/mL), collagenase (0.5 mg/mL), and bromelain (11.75 mg/mL and 6 mg/mL), all dissolved in a balanced salt solution. Various injections (final volume, 200 µL), were prepared and applied centrally into the vitreous body via a thin 27G cannula. In addition, collagenase and hyaluronidase, each with twice the concentration and one-half the volume, were injected as a combination. Control eyes were injected with balanced salt solution. Table presents details on the prepared injections.

**Table.** Details on the Prepared Intravitreal Injections

Substrate	Producer		Material Number/SKU		Concentration (mg/mL)	Volume (mL)
Trypsin from porcine pancreas	Sigma-Aldrich		T7409-1G		1	0.2
Hyal	Sigma-Aldrich		H3506-100MG		0.625	0.2
Coll	Sigma-Aldrich		C1764-50MG		0.5	0.2
Brom	Sigma-Aldrich		B4882-10G		6	0.2

**Combination**	**Injection Procedure**	**Substrates**	**Producer**	**Material Number/SKU**	**Concentration (mg/mL)**	**Volume (mL)**

Combination 1	Coll and Hyal injected	Coll	Sigma-Aldrich	C1764-50MG	1	0.1
(Coll + Hyal)	simultaneously	Hyal	Sigma-Aldrich	H3506-100MG	1.25	0.1
Combination 2	Injection of Brom 24 h	Hyal	Sigma-Aldrich	H3506-100MG	1.25	0.1
(Hyal + Brom)	after the injection of Hyal	Brom	Sigma-Aldrich	B4882-10G	12	0.1

Brom, bromelain from pineapple stem; Coll, collagenase from *Clostridium histolyticum*; Hyal, hyaluronidase from bovine testes.

The enzyme concentration was adjusted for all combined experiments to keep the injected volume unchanged while still delivering the same amount of active enzyme to the vitreous body. The eyes were stored at 8°C after injection and then the measurements of forward light scattering were taken after 24 hours. In a group of eyes treated with hyaluronidase a subsequent injection of bromelain with another 24 hours of storage before dissection was conducted. For these measurements, control eyes were also kept at 8°C for 48 hours. Figure 3 illustrates the study procedures. We chose 8°C in line with work from Filas et al.^28^ An exploratory analysis revealed that, in control eyes that were stored at 8°C, forward light scattering was stable over 48 hours. At 37°C, this was not the case, because post mortem degradation processes are accelerated.

### Statistical Analyses

Statistical analyses were performed using GraphPad Prism 10.0.1 (GraphPad Software Inc., San Diego, CA). *P* values of <0.05 were considered statistically significant. Kolmogorov–Smirnov tests were used to assess normality. All data gathered were distributed normally. One-sample *t* tests or unpaired *t* tests were performed, as appropriate. Statistical tests were performed in a hierarchical order. First, only the results of single enzymatic degradation were tested. Combinatory approaches were only tested if the digestion with hyaluronidase showed significant results.

## Results

### The Effect of Single Enzymatic Degradation on Forward Light Scattering

**Figure 4.**
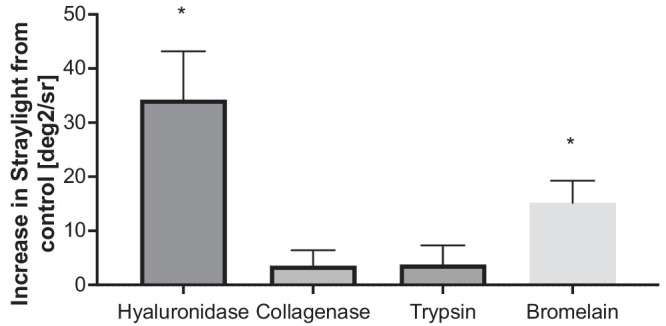
Single enzymatic degradation shows differentiated effects on vitreous gel forward light scattering. Although depleting hyaluronic acid led to a significant increase in straylight, collagenase and trypsin showed no significant effect on the straylight of porcine, juvenile vitreous gels. Bromelain, a previously suggested treatment option for floaters, increased straylight in juvenile vitreous gels. *P = 0.01, baseline straylight values were subtracted from the results of the enzymatically digested vitreous gels, therefore one-sample *t* tests were used. *n* = 6 for hyaluronidase, collagenase and trypsin, *n* = 7 for bromelain.

Enzymatic degradation of the different components of the vitreous gel showed a differentiated effect on forward light scattering. Although enzymatic digestion with trypsin and collagenase did not show a significant impact on the forward light scattering of porcine, juvenile vitreous gels (*P* = 0.28 and *P* = 0.35, one-sample *t* test, respectively), a major increase in straylight was apparent when digesting the vitreous gel with hyaluronidase (*P* = 0.01, one sample *t* test) (Fig. 4).

### Bromelain Increases Forward Light Scattering in Porcine, Juvenile Vitreous Gels

Bromelain was previously tested as an oral supplement to decrease vitreous body opacifications. Thus, we tested the effect of bromelain on vitreous straylight. We tested two concentrations of bromelain (6.00 and 11.75 mg/mL). Owing to their similar effect, results were grouped. When injected into the juvenile vitreous gel, it significantly increased straylight (*P* = 0.01, one-sample *t* test) (Fig. 4).

### Digesting Collagen in Hyaluronic Acid–depleted Vitreous Gels Lowers Straylight Significantly

**Figure 5.**
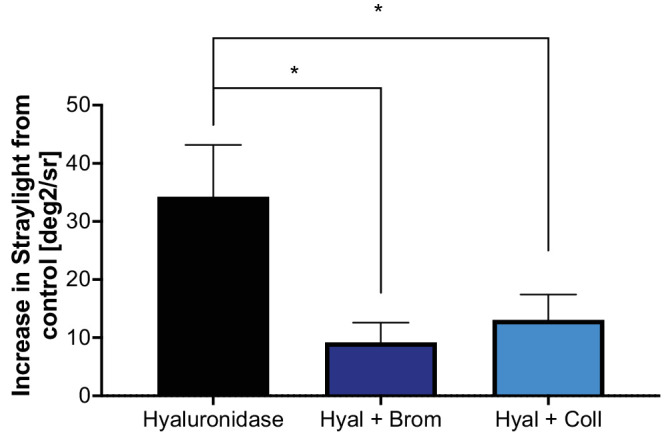
Collagenase and bromelain decrease straylight in hyaluronic acid (Hyal) depleted vitreous gels. When applying collagenase or bromelain to vitreous gels digested with hyaluronidase, a statistically significant decrease in forward light scattering occurred. *P < 0.05 using unpaired *t* tests, *n* = 6 for hyaluronidase and *n* = 8 for both combinatory approaches. The two combinatory approaches were not statistically compared.

As the previous results of the present study showed, depletion of hyaluronic acid causes an increase in straylight. Previously, floaters were identified as conglomerates of collagen and patients with floaters showed increased straylight values.^29^ Thus, for the following experiments, all vitreous gels were digested with hyaluronidase and another agent supposed to break up straylight-inducing collagen conglomerates, namely, collagenase and bromelain. Although collagenase showed no effect on straylight when applied alone, the digestion of collagen significantly decreased the straylight values in hyaluronic acid–depleted vitreous gel samples. Similar results were apparent for the cysteine protease bromelain (Fig. 5).

## Discussion

In this study, we demonstrated that in vitro digestion of the vitreous gel with a variety of enzymes resulted in differentiated alterations of forward light scattering. To objectively measure straylight, we used a previously developed method by our group using a modified C-Quant setup.^25^^–^^27^ Depletion of hyaluronic acid with hyaluronidase lead to a significant increase of straylight, most likely owing to the emergence of collagen conglomerates, whereas collagenase and trypsin did not affect straylight. However, the effect of hyaluronidase was partially reversed after the subsequent application of collagenase or bromelain.

Increased straylight can lead to various symptoms, such as glare or hazy vision, and could, therefore, serve as a useful objective parameter to further evaluate the optical system of the posterior segment. Previously, the measurement of forward light scattering has been used solely to estimate the optical impact of intraocular lens complications such as calcifications or glistenings from different hydrophilic and hydrophobic materials.^25^^,^^27^ Recently, we applied the method for the first time in hydrogels and materials of posterior segment surgery.^30^ To our knowledge, no previous studies have investigated forward light scattering in the enzymatically digested vitreous gel.

The etiology of eye floaters is believed to relate to changes in the molecular structure of the vitreous body. When the hyaluronic acid dissociates from the collagen fibrils, two processes occur: pools of liquified gel, known as lacunae, and conglomerates of collagen fibrils, which are not separated from each other anymore, are formed.^1^ As a result, the vitreous body becomes more inhomogeneous and causes light to scatter and diffract owing to a decreased interfibrillar distance.^31^ In this study, we observed that only the application of hyaluronidase initiated this optical deterioration, leading to the greatest increase in straylight. This finding is also represented in other studies dealing with the viscoelastic properties and the adhesivity of the vitreous gel after enzymatic degradation.^28^ Filas et al.^28^ showed that digesting the vitreous gel with either trypsin, collagenase, or hyaluronidase led to a loss of stiffness of the vitreous gel. However, only the enzymatic digestion of hyaluronic acid increased the adhesivity of the vitreous; this is not the case for vitreous gels treated with trypsin or collagenase. The authors attribute this increased adhesivity to the concentration of collagen and other proteoglycans in the remaining vitreous gel after the loss of water and hyaluronic acid. Similarly, it is reasonable to conclude that the increase in straylight is due to this previously mentioned decreased interfibrillar distance. Both this study and earlier reports, such as the one from Filas et al.,^28^ examined the gel portion of the vitreous body after enzymatic degradation. However, the aged vitreous body is known to consist of a gel and a liquid portion. Owing to the limitations of in vitro forward light scattering measurements, in situ measurements are not feasible currently, and the vitreous body thus must be removed from its cavity, causing a loss of the liquid portion. It is likely that, in vivo, the lacunae, which again contain collagen aggregates, contribute to the increase in straylight. Previously, one clinical study linked the occurrence of symptomatic floaters to forward light scattering. Castilla-Marty et al.^29^ recruited 15 patients with unilateral complaints of floaters. The average straylight value in the symptomatic eyes was increased by a comparable mean value of about 15 deg^2^/sr compared with the fellow eye of the patient used as the reference. This finding supports the thesis that increased straylight is linked to symptomatic floaters. In the present laboratory study, we showed an even greater increase in straylight induced by digesting hyaluronic acid. Our results are within the in vivo range and, thus, could represent severe cases of vitreous opacification.

Here, we also tested the impact the previously suggested pharmacological treatment bromelain. Based on a publication by Ma et al.,^22^ bromelain as a possible treatment for floaters and vitreous opacification gained great Internet popularity. Bromelain is a cysteine protease and theoretically able to digest the collagen making up vitreous opacifications. However, there are no data on its concentration in the vitreous body after being taken as an oral supplement and the study did not include a control group. In the present study, we injected bromelain in concentrations higher than typically reached in serum levels, circumventing the blood–retina barrier. Although we saw a theoretical decrease of straylight values in vitreous gels previously treated with hyaluronidase, the nonspecific enzymatic approach seems questionable, especially regarding collateral damage. Further in vivo studies should elucidate possible cytotoxic side effects.

In this study, we established a direct link between alterations in vitreous components and the manifestation of increased straylight values, providing valuable insights into the mechanisms behind vitreous opacification and at the same time not relying on patients’ reports or semiquantitative analyses. Vitreous gels previously treated with hyaluronidase could offer a simple platform to rapidly assess potential pharmacological interventions, such as enzymatic treatments like bromelain.^22^ Additionally, combinatory enzymatic treatments, including ocriplasmin,^32^ could serve as an accelerated vitreous body aging model to study vitreous opacification in vivo. Previously, such enzymatic treatments have not been evaluated for floater therapy, but mostly for diseases of the vitreoretinal surface, such as macular holes, vitreomacular traction or incomplete, symptomatic posterior vitreous detachment,^33^ or, in cases of hyaluronidase, for vitreous hemorrhage.^34^

### Limitations

Of course, this model holds inherent limitations and only partially imitates the complex process of vitreous body aging and only examines the vitreous gel portion. Posterior vitreous detachment is a common occurrence in old age,^35^ reported to be present in 65% of patients >65 years of age,^36^ and occurs in most patients with symptomatic floaters.^29^ Indeed, it is the most common etiology of floaters.^31^ The purpose of this in vitro model is not to substitute for well-conducted clinical studies, but rather grant researchers the opportunity to rapidly test treatment options. In vivo, the degradation of the vitreous body is predominantly caused by the half-lives of the components and not by active degradation.^2^ In addition, when testing the therapeutic use of the enzymes, this model does not consider possible cytotoxic side effects. Finally, in this study we used porcine and not human vitreous gels because of the greater supply of juvenile porcine vitreous gels with the same age that have no compromised vitreous structure.

## Conclusions

The decline in hyaluronic acid owing to aging is the main factor for the rise in straylight within the vitreous gel, which is caused by altered light scattering and diffraction of improperly spaced collagen conglomerates. Digesting collagen can partially reverse this effect. This in vitro model can be used as a first fast method for testing pharmacological treatments. In the future, this approach should be considered and tested as an in vivo model for vitreous opacifications.
